# Perceived control as a resilience factor: associations with neural, physiological and affective stress responses and mental health

**DOI:** 10.1038/s41398-025-03786-6

**Published:** 2026-01-15

**Authors:** Jana Meier, Bianca Kollmann, Laura E. Meine, Benjamin Meyer, Kenneth Yuen, Magdalena Stork, Oliver Tüscher, Michèle Wessa

**Affiliations:** 1 Leibniz Institute for Resilience Research (LIR), Mainz, Germany; 2 Central Institute of Mental Health, Neuropsychology and Psychological Resilience Research, Mannheim, Germany; 3 Experimental Psychopathology and Psychotherapy, Department of Psychology, University of Zurich, Zurich, Switzerland; 4 Department of Adult Psychiatry and Psychotherapy, Psychiatric University Clinic Zurich and University of Zurich, Zurich, Switzerland; 5 Neuroimaging Center (NIC), Focus Program Translational Neuroscience (FTN), Johannes Gutenberg University Medical Center Mainz, Mainz, Germany; 6 Department of Clinical Psychology and Neuropsychology, Institute for Psychology, Johannes Gutenberg University Mainz, Mainz, Germany; 7 Department of Psychiatry and Psychotherapy, Johannes Gutenberg University Medical Center Mainz, Mainz, Germany; 8 Department of Psychiatry, Psychotherapy and Psychosomatic Medicine, University Medicine Halle, Martin-Luther University Halle-Wittenberg, Halle, Germany; 9 German Center for Mental Health (DZPG), partner site Halle-Jena-Magdeburg, Halle, Germany; 10 DKFZ-Hector Cancer Institute at the University Medical Center Mannheim, Mannheim, Germany; 11 German Cancer Research Center, Division Cancer Survivorship and Psychological Resilience (C160), Heidelberg, Germany; 12 German Center for Mental Health (DZPG), partner site Mannheim-Heidelberg-Ulm, Mannheim, Germany

**Keywords:** Neuroscience, Human behaviour

## Abstract

Perceived control is a key mechanism implicated in stress resilience. A tendency to perceive control over stressors may protect individuals against negative outcomes across various situations by increasing active coping and preventing exacerbated stress reactions. Assuming that individual differences in perceived control during an uncontrollable stress task may represent an underlying resilience factor, we investigated associations of perceived control with neural, endocrine, and affective responses to a different, psychosocial stressor, and with overall mental health. 116 male participants aged 18–30 completed a psychosocial stress task, and we assessed stress responses via functional magnetic resonance imaging, cortisol levels, and affective state questionnaires. General mental health was assessed via self-report. Perceived control was measured during a second, uncontrollable stress task and growth mixture modeling revealed a high- and a low-control class. Comparison of these classes showed that the high-control class experienced less helplessness during the uncontrollability task and demonstrated more flexible responses to psychosocial stress as reflected in cortisol secretion and activation of the bilateral posterior insula. Further, the high-control class reported fewer psychosomatic symptoms and a less external locus of control. These findings suggest that perceived control might act as a resilience factor, influencing stress processing across multiple domains. The study highlights the potential for perceived control to be harnessed in resilience-building interventions and underscores the need for further experimental and longitudinal research to confirm its role in modulating stress responses.

## Introduction

Stress-related psychological disorders are a significant public health issue, yet many people do not develop psychopathology after traumatic events but rather show resilience [1]. Unraveling the mechanisms that drive such resilience could help to prevent stress-related disorders. Perceived control is a mechanism that has been implicated in building resilience by several psychological theories. The transactional stress model [2] and the component process model of emotion [3] suggest that appraisals of control in aversive or threatening situations lead to reduced negative affective reactions. These reactions are linked to stress-related disorders according to the PASTOR model of resilience [4]. This model posits that non-negative appraisals, including high controllability, lead to stress resilience by reducing negative stress reactions. Evidence shows that perceived control over negative life events correlates with better mental health outcomes, including reduced depression and increased well-being [5, 6]. Furthermore, higher subjective control predicted increased longevity in a longitudinal study [7] and has been found to be related to reduced cortisol responses to social stress [8]. Hence, beneficial effects of perceived control might be mediated by a control-dependent reduction of stress reactions, and a better understanding of the physical, neural, and psychological pathways mediating the protective effect of control could help to harness its potential as a resilience mechanism.

The association of control over stressors and stress reactions has been studied in human subjects, as well as in animals. Tasks that include elements of uncontrollability induce strong increases in cortisol in humans, indicating that a lack of control is especially stressful [9]. On a neural level, uncontrollable social-evaluative threat is associated with widespread activation in regions of the salience network, including the fronto-insular cortex [10, 11]. Rodent studies indicate that the protective effect of control over stressors is mediated by the ventromedial prefrontal cortex (vmPFC) that down-regulates stress-related brain regions in the presence of control (for review, see Maier & Seligman [12]). Human neuroimaging studies as well show increased vmPFC activity and decreased activation in stress-related brain areas when stressors are controllable. For instance, higher BOLD responses in the vmPFC and lower responses in the insula were observed when participants controlled mild electric shocks and white noise [13]. Similarly, controllable videos with phobic content elicited more vmPFC and less amygdala activation than uncontrollable videos [14]. Perceived control over nociceptive stimuli reduced activation in pain-related areas such as the anterior cingulate cortex, the insula, and the somatosensory cortex [15]. In sum, human and rodent studies alike indicate that the vmPFC mediates the protective effect of control by inhibiting stress-related regions such as the insula.

However, rodent studies suggest that control has not only immediate effects on stress processing but also strengthens the connection of the vmPFC with stress-related areas, leading to an immunization effect where even uncontrollable stressors no longer hyper-activate the neural stress system. In fact, animals immunized by repeated experiences of control respond to uncontrollable stressors like to controllable ones [16], indicating that they *perceive* uncontrollable situations as controllable. Findings on such trans-situational effects of stressor controllability in humans are mixed. In line with the notion that perceived control may represent stress immunization, internal locus of control (LoC) – the generalized belief that outcomes are controlled by onself [17] – is cross-culturally associated with better mental health [18]. One behavioral study observed increased escape efficiency in a task following controllable aversive stimuli, indicating as well that transfer effects of control exist in humans [19]. In a neuroimaging study, it was possible to classify participants as having experienced uncontrollable or controllable stress by their dorsal anterior insula response patterns during a second, uncontrollable task, showing that control over stressors altered subsequent neural stress processing [20]. Another study found that objective control over electric shocks did not affect later working memory performance [21], but subjective ratings of control did, and also predicted neural processing in prefrontal regions and the left insula. In contrast, a study tested the influence of instrumental control over physical stimuli on the processing of social stress but found only marginal differences in vmPFC connectivity [22]. Hence, there is some evidence that perceived control may generalize and affect multilevel reactions to other stressors, but little is known about the exact underlying processes.

This study aimed to relate perceived control in an uncontrollable stress task as a proxy for stress immunization to multilevel stress responses in another stress task. We intended to build on previous results where we employed a stressor manipulation task with a triadic design and identified latent classes of participants differing in perceived controllability [23]. In the present study, a sample of healthy participants completed an uncontrollable version of this task (“uncontrollability task”) and latent classes of perceived control were similarly derived. Before the uncontrollability task, participants also completed mental health questionnaires and performed a psychosocial stress task while we measured their brain activity with functional magnetic resonance imaging (fMRI), acquired saliva cortisol, and assessed affective stress reactions via self-report. We chose the ScanSTRESS-C as the additional, psychosocial stress task, as it combines different elements that ensure stress reactions in humans like social-evaluative threat and performance pressure, but most importantly, uncontrollability [10]. The compact version [11] was used to reduce scanning time. We decided to conduct the uncontrollability task after the psychosocial stress task, to preclude that the uncontrollability task directly influenced the processing of the psychosocial stressor as this has been described in rodent experiments [24]. Rather than investigating direct transfer effects of control, we assumed that the perceived control classes reflect a tendency to perceive more or less control over stressors that this may also be related to more resilient stress processing.

Hence, the goals of the present study were threefold: First, we aimed to replicate previous findings that the compact version of the psychosocial stress task ScanSTRESS is effective in inducing an affective, endocrine, and neural stress response. Specifically, we expected to see, like in previous studies [11, 25] a marked activation of the HPA axis in a majority of the sample, more negative affect, and an increased neural response in areas of the salience network as well as a downregulation of the default mode network. Second, we expected to see similar latent trajectories of perceived control during an uncontrollability task as well as similar associations with helplessness and affect as we have found in a previous study [23]. And third, we aimed to bring these two tasks together and study differences in responses to the ScanSTRESS-C between participants with high or low perceived control assessed during the uncontrollability task. We expected that the neural and endocrine stress responses in the ScanSTRESS-C would be reduced in participants with higher perceived control as indicated by reduced salivary cortisol and reduced stress-related activation e.g. in the anterior insula in response to psychosocial stress. Building on earlier findings [13], we hypothesize that the protective effect of control may be mediated by a stronger activation of the vmPFC despite uncontrollable stress. Finally, as we hypothesized that perceiving control over stressors generally protects individuals from adverse effects of stress, we expected classes with higher perceived control to report less psychological distress and better mental health.

## Methods and materials

### Participants and procedure

We recruited 120 male participants aged 18–40 via flyers, university postings, social media, and the local residency registration office. The study included only male participants due to several reasons. First, the uncontrollability task is a translation of the learned helplessness task for rodents which is not successful in female rodents [26] and gender differences in response to the task have been found in humans [23]. As we were interested in individual differences in perceived controllability and its effects on different outcome levels, including females would have required twice the sample size to systematically investigate gender effects as well as individual differences with sufficient statistical power. Furthermore, saliva cortisol was measured and sex hormones are known to interact with cortisol responses in laboratory paradigms [27] and controlling for levels of sex hormones and cycle is necessary in studies including women. Given the multi-day paradigm of the current study, it would have been extremely difficult to assure that female participants attended the appointments all in a specific phase of their menstrual cycle. Other exclusion criteria comprised psychiatric or neurological diagnoses, MRI contraindications, beta blocker or psychiatric medication intake, BMI > 27, and studies in psychology or medical fields to avoid prior knowledge of stress induction paradigms that might compromise the effectiveness of the ScanSTRESS-C. Four participants were excluded from data analysis due to a disturbance during the first appointment (1), missing data (1) or dropout (2), resulting in a final sample of *N* = 116.

**Fig. 1:**
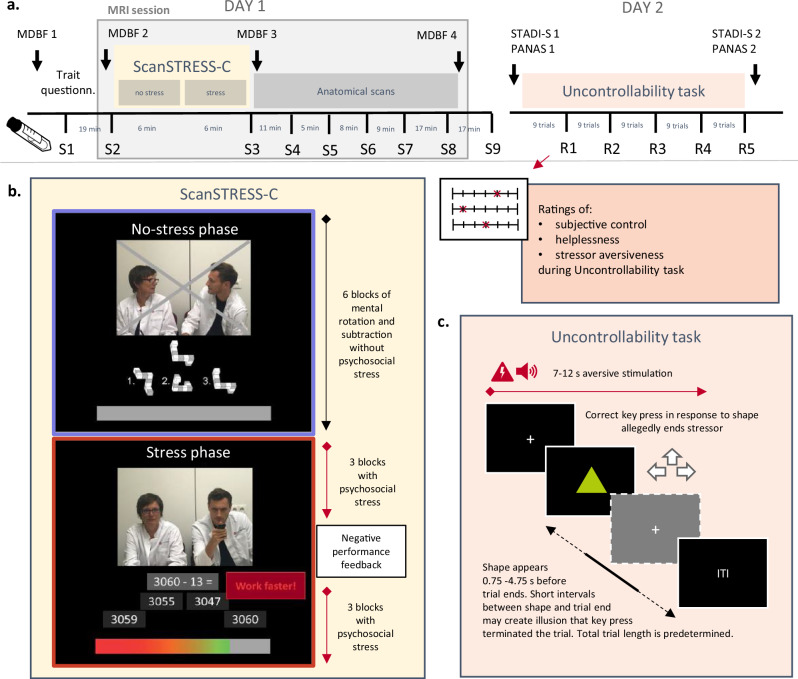
*Experimental procedure.* **a** Overview of the experimental procedure with MRI session including the psychosocial stress task ScanSTRESS-C on day 1 and the uncontrollability task on day 2. **b** ScanSTRESS-C with demonstration of the two cognitive tasks that had to be solved (mental rotation and subtraction) in noStress and stress blocks. Jury not watching in the noStress phase but watching and criticizing slow and inaccurate performance in stress blocks, additional time limit indicated by the colored bar. **c** Uncontrollability task with aversive stimulation that is supposedly terminated by correct arrow key presses in response to a geometric shape. Trial termination is unrelated to key presses, but variable trial lengths can create the illusion of control over stimulation. S1-S9: Saliva samples; R1-R5: Ratings of subjective experience; MDBF: multidimensional mood questionnaire; STADI-S: State-trait anxiety and depression inventory state version; PANAS: positive and negative affect scale, ITI: Inter trial interval. **b** Reprinted and adapted from *Psychoneuroendocrinology*, 118, Sandner, M., Lois, G., Streit, F., Zeier, P., Kirsch, P., Wüst, S., & Wessa, M., Investigating individual stress reactivity: High hair cortisol predicts lower acute stress responses. 104660, Copyright Elsevier Ltd. (2020), with permission from Elsevier.

Participants attended three test appointments within ten days, the first two reported on here. The first appointment included an MRI measurement with the psychosocial stress task (ScanSTRESS-C [11]) and simultaneous saliva cortisol, electrocardiogram, and skin conductance measurements. The latter two are not within the scope of this article, just as further MRI measurements. To control for elevated morning cortisol levels and diurnal variations, MRI sessions started between 11 and 11:30 am. Prior to the ScanSTRESS-C, participants completed a urine drug screen (SureStep^TM^, Diagnostik Nord GmbH, Schwerin, Germany) and underwent a 45 min cool down phase during which they completed trait questionnaires and remained seated to avoid orthostatic effects on cortisol secretion [28]. On the second testing day, participants underwent an uncontrollable stress task, adapted from Meine et al. [19], during which perceived control was assessed at five time points. See Fig. 1a for an overview of the procedure.

Participants received a complete study description, gave written informed consent, and were monetarily compensated. A cover story minimized bias by suggesting the study’s purpose was to measure brain activity during high cognitive performance. Participants were debriefed after the completion of the last test appointment. The study adhered to the declaration of Helsinki and was approved by the local ethical committee of Rhineland-Palatinate (ethics proposal 2018-13270).

### Psychosocial stress task: ScanSTRESS-C

During the first appointment, participants completed the ScanSTRESS-C [11], an adapted version of the ScanSTRESS task [10]. The paradigm consisted of 6 blocks without stress (noStress) serving as baseline, followed by six stress condition blocks. In the noStress condition, participants solved mental rotation and mathematical subtraction tasks with automated performance feedback. During the stress condition, participants faced the same tasks under time constraints and while receiving social evaluative feedback via an online webcam, broadcasting a live jury of so-called experts rating their performance through visual feedback. Response time windows were adapted to each participant’s task performance. After three blocks of stress, the scanner was paused and participants received verbal feedback from the jury via the intercom, telling them to improve their performance, increasing psychosocial stress in the stress condition (see Fig. 1b and Sandner et al. [11] for details). The task ran on Presentation® software version 20.1 (Neurobehavioral Systems, Inc., Berkeley, CA, USA; www.neurobs.com). Participants underwent a short training of the task prior to scanning using simpler task examples without psychosocial stress on a laptop outside the scanner to ensure instruction comprehension.

Affective reactions to the ScanSTRESS-C were measured using the German Multidimensional Mood Questionnaire [29] (MDBF) at four time points during the first testing day. This 24-item questionnaire assesses good/bad mood, alertness/tiredness, and calmness/restlessness. Subscale scores were computed according to the manual.

### Saliva cortisol

Nine saliva samples were collected throughout the MRI session by passive drooling into Salivettes® (Sarstedt AG, Nümbrecht, Germany). The first (−25 min relative to stressor onset) and last ( + 73 min) sample were taken outside of the scanner. The other seven samples were taken at roughly −6, + 6, + 17, + 22, + 30, + 39, and + 56 min relative to stressor onset. The Salivette was placed into the participant’s mouth by the experimenter and rested under the tongue for two minutes. All collected saliva samples were frozen and stored at −20 °C and sent in one batch on dry ice for analyses to the Institute of Biopsychology, Technical University Dresden, Germany. After thawing, samples were centrifuged at 3000 rpm for 5 min, which resulted in a clear supernatant of low viscosity. Salivary concentrations were measured using commercially available chemiluminescence immunoassays with high sensitivity (Tecan-IBL International, Hamburg, Germany). The intra- and interassay coefficients of variance were below 9%.

### fMRI acquisition and analysis

Images were acquired on a 3 T Siemens Trio (Siemens, Erlangen, Germany) using a 32-channel head-coil. Functional T2*-weighted images were obtained with a multiband echo planar imaging sequence with an acceleration factor of 4, 60 slices per run, and the following parameters: TR = 1000 ms, TE = 29 ms, flip angle = 56°, voxel size = 2.5 mm isotropic, FOV = 220 mm. Slices were measured in interleaved order. In addition, T1-weighted MPRAGE images were acquired (TR = 1900ms, TE = 2.52 ms, flip angle = 9°, voxel size 1 mm isotropic, FOV = 250 mm). The task consisted of three runs, i.e., a condition without stress (noStress) serving as baseline and two stress runs, which were separated by oral instructions to improve performance. Data were preprocessed and statistically analyzed using SPM 12 (Wellcome Center for Human Neuroimaging, London, UK) running on Matlab 2017b (The Mathworks Inc., Natick, Massachusetts, USA). Fourteen participants had to be excluded from the neuroimaging analyses due to head movement (>2.5 mm, *n* = 8) and incomplete data (*n* = 6), resulting in a final MRI sample of *N* = 102. To account for scanner equilibrium effects, the first four images of each run were discarded. Images were reoriented to the SPM T1-template to ensure correct orientation of AC/PC in all participants. Movement artifacts were corrected by spatially realigning the functional images to the first image using a six-parameter rigid body transformation. Realigned images were then co-registered to the participant’s individual structural image in native space. Estimated parameters from forward deformations resulting from segmentation of participant’s structural image, were used to normalize images into the Montreal Neurological Institute (MNI) standard space. During normalization, images were resampled to 2 mm isotropic and finally smoothed using a Gaussian kernel of 8 mm full width at half maximum.

First-level analyses used a general linear model with noStress and Stress regressors, modelling the onsets of the respective blocks with a fixed duration of 40 and 20 s pause in-between blocks, plus six motion regressors. Main contrasts were Stress > noStress and noStress > Stress.

### Uncontrollability task

At the second study appointment, participants completed a task adapted from Meine et al. [19]. It included stressful stimulation with white noise bursts via headphones (Sennheiser HD 380 Pro, Sennheiser, Wedemark, Germany) at 85 dB and aversive electric shocks administered through a wasp electrode attached to the back of the left hand and produced by a Digitimer DS7A current stimulator (Digitimer Ltd, Welwyn Garden City, UK). Electric shock intensity was calibrated for each participant individually to be unpleasant, yet not painful. The perceptual threshold was determined, and shock intensity increased in steps of 0.5 mA, until a rating of *unpleasant* to *very unpleasant* was reached, corresponding to 4–5 on a scale from 1 (*barely noticeable*) to 10 (*extremely painful*).

The task included 45 trials, each starting with a fixation cross, followed after 1 s by stressful stimulation in the form of concurrent double shocks 20 ms apart, every 1.5 s +/−0.25 s and white noise bursts divided by short breaks of 50–200 ms. After a variable time interval (see below), a green triangle, square or circle was presented on screen. Participants were told that each shape was assigned to one of three designated arrow keys (up, left, or right) on a keyboard and they would be able to stop the aversive stimulation by figuring out the shape-key assignment and pressing the correct key fast enough. In reality, the total duration of stimulation in each trial was predetermined: Each participant completed the same randomized sequence of preset trial durations ranging from 7.13 to 11.38 s (*M* = 9.12, *SD* = 1.16). The shape was presented at a random time point uniformly distributed between 4.75 to 0.75 s before the end of the trial. Consequently, the trial sometimes ended shortly after the shape was presented, which could give participants the impression that they terminated the stressor by their key press, while in other trials, the aversive stimulation continued. Each trial was followed by an intertrial interval (ITI) of 3 s +/−0.25 s.

Unpleasantness, perceived helplessness, and control were rated after every ninth trial on a 7-point Likert scale from *not at all* to *very much*. State anxiety, depression, and negative affect were assessed before and after the task using the German versions of the state-trait-anxiety-depression inventory - state (STADI-S [30]), and positive negative affect schedule (PANAS [31]). We obtained sum scores as described in the test manuals and computed difference scores by subtracting pre from post scores.

### Self-report measures

Mental health over the last couple of weeks was measured with the General Health Questionnaire German Version (GHQ-28 [32, 33]), assessing somatic symptoms, anxiety, social dysfunction, and depression. Higher scores indicate worse mental health. Participants also completed the German versions of the IE-4, a short instrument assessing internal and external locus of control [34] and the General Self-Efficacy Scale (GSE [35]).

### Statistical analyses

Analyses of the behavioral and endocrine data were performed in R version 4.3.3 [36] running in RStudio [37]. The significance level was set to 0.05, tests were two-sided and p-values were corrected for multiple comparisons. For repeated measurements ANOVA (rmANOVA), Greenhouse-Geisser correction was applied when sphericity assumptions were violated.

### Analysis of perceived control: growth mixture modeling

To investigate individual differences in subjective control, growth mixture models (GMMs) were estimated based on the trajectories of perceived control during the uncontrollability task. One to six class models with random intercepts and slopes were fitted using the package lcmm [38] and resulting models were compared based on the Bayesian Information Criterion (BIC), entropy and theoretical plausibility. Lower BIC and entropy closer to 1 indicate better model fit and discriminant power of the model and entropy greater than 0.7 is acceptable [39]. Classes smaller than 10% of the sample were deemed too small to allow for robust interpretation of the results. Derived class membership was then used as a grouping variable to identify differences in endocrine and neural stress responses to the ScanSTRESS-C task, in affective responses to the uncontrollability task, and in mental health scores. As generalized control beliefs like LoC may be related to the perceived control classes, we additionally examined class-differences in LoC and correlations of LoC with all stress outcomes.

### Analysis of self-report measures

As manipulation checks we assessed the changes in self-reported affect induced by the two tasks in the full sample. For the ScanSTRESS-C, we computed one-way rmANOVAs for good mood, alertness and calmness with the within-subject factor time (4 levels). Greenhouse-Geisser correction was applied if the assumption of sphericity was violated. Post-hoc tests comparing the measurements 2 and 3 before and after the ScanSTRESS-C were computed using the emmeans package [40] and corrected with the Tukey method. For the uncontrollability task, we conducted three paired *t*-tests between pre and post measures of the STADI-S subscales depression and anxiety and the PANAS.

To investigate the effect of perceived control on multilevel stress reactions, we compared the GMM classes in self-report measures of affect and mental health. To that aim, we conducted a MANOVA with the factor class and eight dependent variables: Helplessness, change in state depression, state anxiety and negative affect over the course of the uncontrollability task, and the four subscales of the GHQ-28 (somatic, depression, anxiety, and social dysfunction). Because the assumptions of multivariate normality, as well as covariance homogeneity were violated, the robust rank-based Wilk’s lambda was chosen as test statistic [41]. The MANOVA was followed up with Welch-tests, *p*-values were Holm-corrected for multiple comparisons.

### Analysis of endocrine responses

For the saliva cortisol data, participants were defined as responders if they showed a baseline-to-peak increase of >= 1.5 nmol/l [42]. The success of the ScanSTRESS-C in inducing an endocrine stress response was tested with an rmANOVA on the cortisol measurements with the within-subject factor time (9 measurements). A complementary mixed ANOVA was computed with the additional between-subject factor responder (yes/no) and the interaction time x responder. As measures of total cortisol secretion and increase in response to the stressor, we computed the area under the curve with respect to ground (AUC_g) and with respect to increase (AUC_i), respectively [43]. We only included the time points 2–7 (−6 min, + 39 min) for these measures as saliva cortisol typically peaks 20–30 min after onset of psychosocial stress [11, 44]. A recovery index was computed as the difference between individual peaks and the last measurement (+73 min). To assess differences in cortisol response to the ScanSTRESS-C between the perceived control classes, we computed a MANOVA with the dependent variables AUC_i, AUC_g, and recovery index and the factors class and cortisol responder (yes/no). Again, the assumption of normality was violated but the covariance matrices were homogeneous. As MANOVA using Pillai’s Trace is rather robust against violations of the distributional assumptions when covariance matrices are homogeneous [45], Pillai’s trace was chosen as test statistic. The MANOVA was followed up by separate ANOVAs that were Holm-corrected for multiple comparisons.

### Analysis of neural data

For the second-level analyses of the neuroimaging data, a one-sample *t*-test was performed to determine the acute stress induction’s effect (i.e. Stress > noStress, noStress > Stress). The significance level was set to *p* = 0.05, whole-brain family-wise error (FWE) corrected at voxel level. To assess class-differences in the neural stress response, the contrast images Stress > noStress were entered into a two-sample *t*-test comparing the two classes. We used a statistical threshold of *p* = 0.05, FWE corrected at the cluster level, with a cluster forming threshold of uncorrected *p* = 0.001. Individual parameter estimates for the peak voxels of significant clusters were extracted with a custom Matlab script for the Stress and noStress regressors. As previous findings point to the vmPFC to be a critical structure for stressor controllability effects in animals [12] and humans [13], we conducted an additional region of interest (ROI) analysis restricted to the vmPFC. The coordinates for the ROI were derived from the peak coordinates (−6 36 −8) active during controllable vs. uncontrollable stress in an MRI compatible version of the uncontrollability task [13]. The ROI was created based on an approach described by previous investigators for the medial PFC [46, 47] by setting x = 0 to center it and constructing a box of 16 × 20 × 20 mm around it. We applied small-volume correction (SVC) at *p* = 0.05, FWE-corrected at voxel level.

## Results

### Manipulation checks

#### Affective stress response

For affective responses to the ScanSTRESS-C, rmANOVAs revealed a significant effect of time on positive mood, *F*(2.41,270.36) = 91.21, *p* < 0.001, η^2^g = 0.21, alertness *F*(1.95,218.03) = 33.15, *p* < 0.001, η^2^g = 0.08, and calmness, *F*(2.52,282.73) = 134.721, *p* < 0.001, η^2^g = 0.29. Post-hoc tests showed a significant decrease from measurement 2 to 3 (pre vs. post stress intervention) in good mood (*p* < 0.001), alertness (*p* = 0.003), and calmness (*p* < 0.001; see Figure S1a-c).

For the affective responses to the uncontrollability task, paired t-tests revealed a significant increase from pre to post for state depression, *t*(115) = −8.65, *p* < 0.001, for state anxiety, *t*(115) = −7.43, *p* < 0.001 and for negative affect, *t*(115) = −6.30, *p* < 0.001 (see Figure S1e-g).

#### Cortisol response

For the analysis of endocrine responses to the ScanSTRESS-C, 18 participants had to be excluded due to insufficient saliva sample volumes. The rmANOVA on the cortisol measurements showed a significant effect of time, *F*(2.9,243.71) = 21.49, *p* < 0.001, η²g = 0.12. Post-hoc tests between all adjacent measurements revealed a significant increase from measurement 3 to 4 (*p* < 0.001) and a trend for a decrease from measurement 5 to 6 (*p* = 0.054), indicating that in general, the sample showed an endocrine stress response to the task. However, as is common in psychosocial stress tasks [44], some participants did not show an increase in salivary cortisol: Only 63.3% of the sample were classified as responders. We conducted an additional mixed ANOVA with the added between-subject factor responder that showed significant effects for responder, *F*(1,83) = 25.54, *p* < 0.001, η²g = 0.13, time, *F*(3.63,301.18) = 12.52, *p* < 0.001, η²g = 0.07, and the interaction responder*time, *F*(3.63,301.18) = 18.36, *p* < 0.001, η²g = 0.10. Post-hoc tests revealed that, for non-responders, adjacent measurements did not differ significantly. For responders, there was a significant rise in cortisol from measurement 3 to 4 (*p* < 0.001) and significant decreases from measurements 5 to 6 (*p* = 0.005), 7 to 8 (*p* = 0.017) and 8 to 9 (*p* = 0.018), see Figure S1d. Hence, the ScanSTRESS-C successfully induced activation of the hypothalamic-pituitary-adrenal (HPA) axis in responders.

#### Neural stress response

The main effect Stress > noStress showed activations in regions related to the executive control network, including the middle frontal gyrus, inferior parietal lobule and dorsal precuneus, and of the salience network, most notably the anterior insula. Moreover, the supplementary motor area and widespread parts of the occipital lobe were activated.

The reversed contrast noStress > Stress revealed activations in regions of the default mode network, such as the posterior cingulate cortex, and medial frontal cortex, but also sensorimotor cortices, the cerebellum, posterior insula and the striatum. Please refer to Table S1 and Figure S2 in the supplement for details.

### Perceived control trajectories: growth mixture modeling

**Fig. 2:**
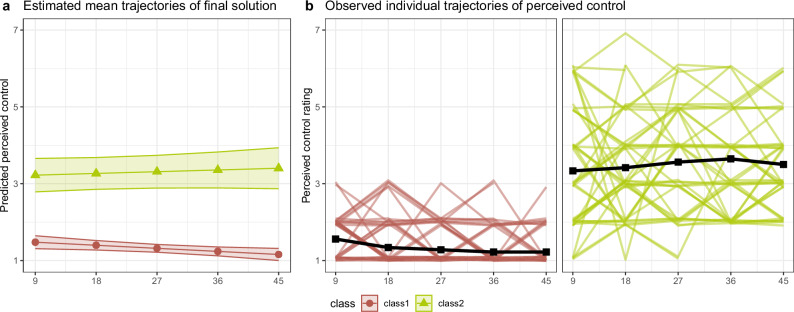
*Predicted and observed trajectories of perceived control of the latent classes identified with GMM.* The best-fitting growth mixture model was a model with two latent classes. Estimated **a** and observed **b** trajectories of these classes differed in mean level of perceived control, resulting in a low-control and a high-control class. The low-control class had a negative slope, indicating that perceived control decreased even more over time for these participants.

To identify latent classes representing differing trajectories of perceived control during the uncontrollability task, we fitted GMMs with 1–6 classes on the control rating trajectories. See Table 1 for model comparisons. The 2-class model had the best fit. Predicted and observed trajectories of perceived control of the two classes can be seen in Fig. 2. The bigger class (*n* = 68) had very low perceived control that decreased even more over the course of the uncontrollability task, as indicated by a significant negative slope (*b* = −0.009, *p* = 0.014). The smaller class (*n* = 48) had a higher level of perceived control and a non-significant slope, *b* = 0.005, *p* = 0.454. See Table 2 for descriptive statistics of the derived classes. Although perceived control is likely related to generalized control beliefs like LoC, the classes differed only marginally in LoC and LoC was not statistically related to any of the outcome measures (all *r*s < 0.30, all *p*s > 0.05, see Table S1 for details).

**Table 1:** Model comparison for growth mixture models.

G	Max LL	conv	npm	BIC	Entropy	% participants in classes					
						1	2	3	4	5	6
1	−862.97	1	6	1754.5	1.00	100					
2*	**−814.01**	**1**	**10**	**1675.6**	**0.73**	**58.6**	**41.4**				
3	−807.43	1	14	1681.4	0.79	15.5	57.8	26.7			
4	−806.63	1	18	1698.8	0.77	52.6	15.5	7.8	24.1		
5	−805.95	1	22	1716.5	0.78	7.8	8.6	53.5	25.0	5.2	
6	−800.64	2	16	1724.9	0.84	7.8	5.2	7.8	12.1	51.7	15.5

Model fit for growth mixture models with different numbers of latent classes. The 2-class model had the best fit indicated by the lowest BIC, acceptable entropy and reasonable class sizes.

Model formula: control rating ~ trial, subject = ID, random = ~1 + trial.

G = number of classes, *Max LL* maximum loglikelihood, *conv* converged (1=yes, 2=no), *npm* number of parameters, *BIC* Bayesian Information Criterion.

* chosen model.

**Table 2:** Descriptive statistics for the latent classes.

Variable	Low	High	Full sample	*t/*Χ²	*p*
*N*	68 (58.6%)	48 (41.4%)	116		
Age	26.32 (5.37)	26.60 (5.66)	26.44 (5.47)	−0.27	0.789
Highest educational degree					
None	-	-	-		
Middle school	2 (2.9%)	1 (2.1%)	3 (2.6%)		
High school	29 (42.6%)	13 (27.1%)	42 (36.2%)		
Apprenticeship	9 (13.2%)	10 (20.8%)	19 (16.4%)		
University degree	27 (39.7%)	24 (50%)	51 (44%)		
Not reported	1 (1.5%)	-	1 (0.9%)		
General self-efficacy	30.91 (3.68)	30.15 (3.56)	30.60 (3.64)	1.11	0.271
Locus of control					
internal	4.38 (0.55)	4.36 (0.56)	4.38 (0.56)	0.17	0.866
external	1.97 (0.51)	1.77 (0.65)	1.89 (0.58)	1.85	0.067
Stressor aversiveness	5.64 (0.99)	5.00 (1.20)	5.38 (1.12)	3.04	0.003**
Mean perceived control	1.32 (0.31)	3.49 (1.23)	2.22 (1.35)	−11.93	<0.001***
Shock intensity	3.72 (2.48)	4.29 (2.53)	3.96 (2.51)	−1.20	0.233
Stimulation duration per trial	9.13 (1.16)	9.13 (1.16)	9.13 (1.16)		
Cortisol					
*N*	59 (60.2%)	39 (39.8%)	98		
Cortisol responders	36 (61%)	26 (66.7%)	62 (63.3%)	0.13	0.724

Description of the latent classes derived from growth mixture modeling. The classes did not differ in age or general self-efficacy. External locus of control was higher in the low class with a trend towards significance. The low class reported greater subjective stressor aversiveness, but the classes did not differ in objective shock intensity, indicating that this was not caused by differing shock levels. One participant from the low class had to be excluded from the comparison, as due to technical difficulties, his shock intensity had to be set to the maximum of 99.9 mA. For continuous variables: Mean (SD). For categorical variables: Count (Percent). *T*, Χ² and *p* values derived from *t*-tests and Χ²-test. **p < 0.01, ***p < 0.001.

### Differences in stress responses between the perceived control classes

#### Class differences in self-report measures

The rank-based one-way MANOVA on self-report measures showed a significant effect of class, *X*^2^(8) = 39.24, *p* < 0.001, Wilk’s Λ=0.70. The follow-up Welch-tests revealed that the high-control class reported significantly lower helplessness, *t*(88.69) = 5.51, *p*_Holm_ < 0.001, *d* = 1.06, and fewer somatic symptoms in the GHQ-28, *F*(107.2) = 3.09, *p*_Holm_ = 0.018, *d* = 0.58. The effect of class on negative affect did not survive correction for multiple comparisons, *t*(113.97) = 2.43, *p*_Holm_ = 0.101, *d* = 0.44. There was no effect of class on change in state depression, *t*(109.17) = 0.38, *p*_Holm_ > 0.999, and anxiety, *t*(106.37) = 0.57, *p* > 0.999, in response to the uncontrollability task, or the GHQ-28 subscales depression, *t*(113.99) = 0.68, *p*_Holm_ > 0.999, anxiety, *t*(104.38) = 0.51, *p*_Holm_ > 0.999, and social dysfunction*, t*(100.48) = 0.72, *p*_Holm_ > 0.999 (Figure S2). As the class assignment was probabilistic, there was variability in the certainty with which an individual belonged to the class they were assigned to. To account for this variability, we ran an additional multivariate linear regression analysis analogous to the MANOVA for the outcomes that showed a significant association with class in the MANOVA, i.e. helplessness and the somatic symptoms subscale of the GHQ. Instead of the class assignment, we added as predictor the individual posterior probabilities for belonging to the low-control class (as there were only two classes, the probabilities for the high-control class were the complementary probabilities). There was an effect of the class probability on helplessness, R² = 0.27, *F*(1113) = 41.49, *p* < 0.001, β = 0.51, and somatic symptoms, R² = 0.08, *F*(1113) = 9.60, *p* = 0.003, β = 0.28. Thus, the multiple regression confirmed the results of the MANOVA.

#### Class differences in endocrine stress response

**Fig. 3:**
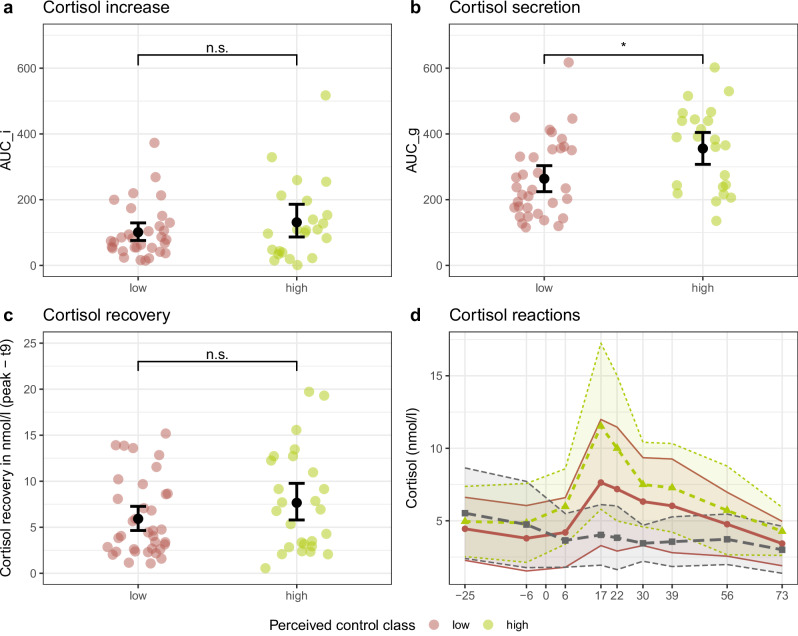
*Class differences in cortisol response to psychosocial stress.* Cortisol increase (**a**), total cortisol secretion (**b**), cortisol recovery (**c**) and mean cortisol reaction (**d**) in response to the ScanSTRESS-C task for the two perceived control classes derived from growth mixture modeling. Non-responders not included in plots a-c, errorbars denote bootstrapped 95% confidence intervals. Margins in (**d**) are standard deviations. AUC_i: Area under the curve with respect to increase, AUC_g: Area under the curve with respect to ground, n.s.: non-significant, * *p*_Holm_ <0.05.

The MANOVA on the cortisol measures revealed a significant effect of class, *F*(3,80) = 3.06, *p* = 0.033, Pillai’s trace=0.10, of responder, *F*(3,80) = 19.98, *p* < 0.001, Pillai’s trace=0.43, and of the interaction class x responder, *F*(3,80) = 2.94, *p* = 0.038, Pillai’s trace=0.10. The follow-up ANOVAs demonstrated that after Holm-correction for multiple comparisons, there was a significant effect of responder for all outcomes (*p*s_corr_ < 0.001) and for the interaction class x responder for AUC_g (*p*_corr_ = 0.036). The post-hoc comparisons following up on the interaction revealed that for responders, the high-control class showed significantly higher cortisol secretion indicated by AUC_g, *T*(82) = −3.26, *p* = 0.002; but there was no class difference in non-responders, *T*(82) = 0.84, *p* = 0.403 (Fig. 3). This was contrary to our hypothesis that perceived control would be related to a lower cortisol response. Again, to account for variability in the probability with which individuals belonged to one or the other class, we ran an additional linear regression analysis with AUC_g as outcome and the predictors class probability and responder. The overall regression was significant, R² = 0.33, *F*(3,82) = 13.46, *p* < 0.001. There was a highly significant effect of responder (β = 1.12, *p* < 0.001) and the interaction responder x class probability approached significance (β = −0.38, *p* = 0.053). While not fully reaching significance, the regression supported the results of the MANOVA.

#### Class differences in neural stress response

**Fig. 4:**
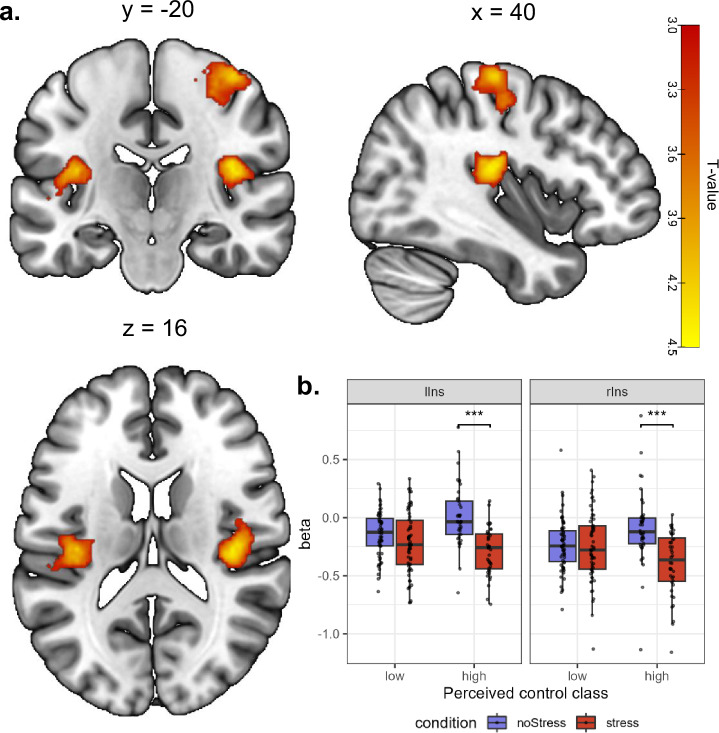
*Class difference in neural response to psychosocial stress.* Significant clusters (**a**) and parameter estimates at peak voxels of posterior insula clusters (**b**) for the comparison of the low and high-class for the contrast stress>noStress. Activation maps are thresholded at p = 0.05 FWE-corrected on cluster-level and overlayed onto the SPM152 template. lIns left insula, rIns right insula, *** p < 0.001.

The 2-sample t-test for the contrast Stress > noStress revealed three clusters that differed significantly between classes in the bilateral posterior insula (PI), R: peak at 40 −20 16, *T* = 4.44, *p*_FWE_ = 0.011, 409 voxels; L: peak at −36 −20 16, *T* = 4.27, *p*_FWE_ = 0.007, 455 voxels, and the postcentral gyrus, peak at 40 −20 62, *T* = 4.28, *p*_FWE_ < 0.001, 777 voxels (Table S2). Parameter estimates indicated that for the high-control class, the activation in the PI was reduced under Stress, compared to noStress, while no such difference between the conditions was observable in the low-control class (see Fig. 4). For the inverse contrast, no activations differed between the classes. As for the behavioral analyses, we ran an additional multiple regression model to account for the uncertainty with which individuals were classified into the classes by GMM, with the individual posterior class probability for the low-control class as regressor. The results were vastly similar, except for a slightly different peak voxel in the left insula and a bigger extend of all clusters, for details, please refer to Table S4 in the supplement. The ROI analysis for the vmPFC showed two clusters that were significantly more activated in the low-control class, compared to the high-control class for the contrast Stress > noStress, cluster 1: peak voxel = 8 32 −16, *T* = 3.37, *p*_*SVC-FWE*_ = 0.037, *k* = 9; cluster 2: peak voxel: −4 42 −12, *T* = 3.30, *p*_*SVC-FWE*_ = 0.043, *k* = 17 (Figure S4 in the supplement). This was contrary to our hypothesis, as we had expected higher vmPFC activation in individuals with higher perceived control under stress. No clusters were significant for the opposite contrast.

## Discussion

The goal of the present study was to identify the influence of perceived control over an uncontrollable aversive event on responses to an independent psychosocial stressor as well as general mental health. To this end, we identified latent classes of individuals based on their control rating during an uncontrollable stress task and compared the obtained classes with respect to multilevel stress responses and mental health. Our results indicate that participants who perceive more control in the uncontrollability task report reduced helplessness during the task but also show a differential neural and endocrine response to a psychosocial stress task and report better mental health.

In line with previous results from our group [23], there was substantial heterogeneity in perceived control in the uncontrollability task and participants could be classified by GMM as belonging to one of two classes, a high- and a low-control class. The high-control class reported lower helplessness in response to the uncontrollability task, mirroring earlier results [23]. This may seem trivial, as a negative correlation between ratings of controllability and helplessness is to be expected. Yet, the substantial, but moderate correlation also indicates that stressor controllability and helplessness do not represent the same construct. Indeed, we believe that helplessness goes beyond a perceived lack of control as it involves attributional processes (“I’m helpless”, as opposed to “this situation is not controllable”). Theoretical work by Abramson et al. building on the findings on stressor controllability in animals emphasized that attributional processes shape the response to uncontrollable stress and posited that feelings of general helplessness could contribute to clinical depression [48, 49]. The high ratings of helplessness in the low-control class indicate that these individuals not only perceived little control over the specific task but may react with helplessness to uncontrollable stress. That a substantial proportion of participants was protected from feeling helpless despite an objectively uncontrollable stressor underlines the relevance of subjective - even illusory - control for resilience. Perceived control may constitute a malleable target for cognitive interventions and prevent stress-related impairments.

Targeting perceived control with adequate interventions is only valuable, however, if the tendency to perceive control over stressors is relatively stable and influences stress responses across different situations. Hence, the main goal of the present study was to show that higher perceived control over a stressor contributes to more adaptive stress responses in an unrelated task. Indeed, we found that the classes identified by their trajectory of perceived control in the uncontrollability task also varied in their reactions during a psychosocial stress task. More precisely, the high-control class showed increased cortisol secretion and reduced neural activation in the PI in response to psychosocial stress. Moreover, the high-control class reported fewer psychosomatic symptoms in the preceding weeks. Taken together, the observed class differences indicate that perceived control may represent an underlying latent variable or resilience factor that seems to be related to stress processing across different situations and domains, aspects of mental health and personality.

Remarkably, self-reported locus of control, which is thought to reflect generalized expectancies regarding control, was only slightly more external in members of the low-control class. Moreover, it was not statistically related to stress outcomes or mental health. These results indicate that internal/external locus of control and perceived controllability in our experiment capture different aspects of perceived control. General control beliefs are moderately stable over time when assessed by questionnaires [50] and are not specific to stressful situations. The control rating, on the other hand, allows to study the effect of experimental manipulations on short-term changes in perceived control in a specific situation. This may give insights into the mechanisms underlying the formation of control beliefs. Furthermore, perceived control over an acute stressor may be more directly related to the processing of this stressor, but also general stress coping tendencies. We thus believe that investigating latent classes of control trajectories and relating them to stress outcomes provides a valuable complement to traditional self-report measures of control beliefs in the study of perceived control as a resilience factor.

To shed more light on the nature of the classes, we examined the observed differences in detail. The higher total cortisol secretion as indicated by the AUC_g in the high-control compared to the low-control class contradicts our hypothesis as well as previous findings where perceived control and a more internal locus of control were related to attenuated cortisol reactions [8, 51]. Yet, another study, in line with ours, found higher perceived control to be associated with higher cortisol increase in response to cognitively demanding tasks [52], indicating a complex association of cortisol, perceived control, and stress. In fact, cortisol modulates stress responses intricately: Basal cortisol levels permit other fast-acting stress hormones like catecholamines to unfold their effect when a stressor occurs, while a stress-induced increase of cortisol prevents overshooting stress reactions and prepares the organism for future stressors [53]. This means that cortisol likely mobilizes resources for active coping but also downregulates the stress response once it is no longer necessary. Dampened HPA axis activity in individuals who perceive little control over stress may impair flexible initiation and termination of coping responses when confronted with stressors.

We observed a similar lack of flexible responding in neural responses to the ScanSTRESS-C: While the high-control class showed deactivation of the PI under stress, compared to the noStress condition, no such stress-related adaptation of PI activation was found in the low-control class. This challenges findings of either no controllability-dependent modulation of PI activation [13, 22], or even increased activation of the PI in response to controllable stress [54]. However, these studies compared controllable to uncontrollable stress, whereas the ScanSTRESS-C contrasts uncontrollable stress with a non-stressful control condition. Hence, the association of the PI and controllability warrants further investigation. Generally, activation of the insula has been linked to stress and pain: The entire insula has been found to be active under threat of social exclusion [55] while the posterior part is implicated in pain processing [56, 57] and direct stimulation of the PI induces sensations of pain [58]. Notably though, the ScanSTRESS-C involves no physical stressors, so the PI activation did not reflect differential sensitivity to noxious stimuli. The variance in PI activation may thus relate to differences in stress processing, which is supported by the observed class difference in cortisol response. Across classes, the PI was generally less activated under stress, mirroring findings of Sandner et al. [11] who found a similar cluster to ours to be deactivated under psychosocial stress. Acute stress reduces perceived pain, an effect that may be mediated by cortisol [59]. For instance, cortisol has been found to reduce pain-related activation of the PI [60]. Consistent with this, individuals in the high control class showed both increased cortisol responses and reduced activation of the PI under stress in the ScanSTRESS-C. Preventive down-regulation of a pain-related area under acute stress may be adaptive as lowered pain-sensitivity may allow an individual to escape from a harmful situation despite an injury. Interestingly, a decreased cortisol reaction to social stress has been longitudinally linked to increased pain sensitivity and musculoskeletal pain [61]. Moreover, attenuated morning cortisol has been found in patients with persistent pain who also reported stronger depressed mood and dysfunctional coping strategies [62]. This is in line with our finding that the low-control class not only showed reduced cortisol secretion but also failed to down-regulate a pain-processing region and reported more psychosomatic symptoms. Hence, individuals who perceive little control over stressors may fail to flexibly respond to stress on endocrine and neural levels and this may manifest over time in psychosomatic distress.

The results of the ROI analysis in the vmPFC contradicted our hypothesis: We observed reduced activation of the vmPFC under stress in the high-control class. The substantial body of research on stressor controllability in animals points to the vmPFC as the structure that detects control and mediates its protective effects [12]. These findings have been corroborated by human neuroimaging studies showing increased activation of the vmPFC during controllable, compared to uncontrollable aversive stimuli [13, 14]. In contrast, some studies have found the vmPFC to be more active in groups experiencing no control over stressor offset [63, 64]. Activation in the vmPFC was found to correlate with increased pain ratings in response to noxious stimuli that were perceived as uncontrollable [65]. As we did not compare controllable to uncontrollable stress and did not assess perceived control during the ScanSTRESS-C, we cannot directly relate the observed difference in vmPFC activation to stressor controllability. The vmPFC has also been implicated in other processes, namely representation of subjective value, (re)appraisal of affective stimuli and social judgements [66]. The increased vmPFC activation in the low-control class may thus be related to affect regulation or the processing of the social-evaluative stimuli, rather than perceived control. It is striking, however, that - like in the insula - the vmPFC activation for the high-control class differed between the conditions of the ScanSTRESS-C, whereas the low-control class showed the same activation for noStress and Stress blocks. This indicates that the low-control class may be less efficient in discriminating safe from unsafe situations. In future studies, perceived control should also be assessed during the ScanSTRESS-C to directly associate it with vmPFC activation and better understand its role in human stress processing.

While our study provides important evidence that perceived control is related to differential stress processing on multiple response levels, it has some important limitations. First, we cannot make claims about the causal direction of the observed effects, as the task order was not randomized and the classes were derived empirically, not experimentally. Hence, we cannot preclude the possibility that the experience of the ScanSTRESS-C differed between individuals in the high- and low-control class and these differences influenced perceptions of control in the uncontrollability task. However, we believe that this is unlikely for several reasons. First, the classes differed not only in perceived control, but also in outcomes across both tasks and in traits that were assessed before the ScanSTRESS-C. Moreover, although transfer effects cannot be ruled out by our design, we believe that such transfer effects from the ScanSTRESS-C to the uncontrollability task over several days from one study appointment to the next are rather unlikely. Second, the ScanSTRESS-C was no less stressful for the high-control class, as indicated by higher cortisol levels. And third, in a previous study [23] where participants completed the uncontrollability task without any other stressful experience preceding it, we have found very similar classes of control trajectories that were likewise associated with helplessness and negative affect in the stressor controllability task (although in the present study the class difference in negative affect did not survive multiple comparisons correction). Thus, although we cannot know whether the class differences in stress outcomes are (fully) attributable to differences in perceived control, we have identified two groups of participants that respond with distinct response patterns to two unrelated stress tasks. Moreover, these groups differ in psychopathological symptoms and control beliefs. Following up our results by investigating systematically whether perceived control is in fact the factor underlying the class differences using different stress tasks, longitudinal study designs and different populations could give valuable insights into adaptive stress reactions and the potential of perceived control as a resilience factor. In sum, we believe that the present study helps to fill a research gap, as previous studies using experimental manipulations without taking perceived controllability into consideration, have struggled to find trans-situational effects of stressor controllability [21, 22]. The classes identified by the uncontrollability task may provide important insights on the systems that are related to perceived control and inform future experimental studies on the outcomes in which controllability-dependent effects can be expected.

Second, our sample consisted only of rather educated, healthy young males, which limits the generalizability of our findings and necessitates replication in more heterogeneous samples. Specifically, the exclusion of female participants is a serious limitation given the higher incidence of some stress-related disorders including depression in women [67] and future studies should investigate the effects of control over social stress in females. Moreover, excluding participants that fulfill diagnostic criteria for any mental disorder limits the generalizability of the findings. However, initiatives like the RDoC framework [68] encourage the definition of mental health as a spectrum. Resilience research further postulates that mechanisms to prevent psychopathology can be studied in healthy samples that still vary with respect to psychological distress. The class difference in psychosomatic symptoms indicates that perceived control could play a role in the development of stress-related impairments that might compromise individuals’ lives beyond full diagnosis of mental disorders. Yet, it would of course be of interest to investigate if classes with differential perceived control also exist in clinical populations with varying psychopathological symptoms.

Finally, future studies should include more and faster-acting stress markers such as, for instance, salivary alpha-amylase [69] to provide valuable information on the interplay of different stress-hormones in relation to perceived control.

## Conclusion

On a cautious note, we assume that the observed class differences are related to an underlying tendency to perceive control over important negative aspects of life. Lower perceived control may be associated with maladaptive changes in stress processing and possibly contribute to impaired mental health. The association between perceived control and endocrine as well as neural stress responses might also indicate different therapeutic interventions: Cognitive-behavioral measures could support individuals in identifying ways of controlling negative aspects of their lives, possibly supported by neurofeedback. Pharmaceuticals or neurostimulation could increase systemic cortisol levels and activation of the insula and in turn influence perceived control from a physical level. However, as our results are purely correlational, experimental and longitudinal studies are needed to confirm perceived control as a resilience factor modulating multilevel stress responses.

## Supplementary information

Table of Contents of Supplementary Information

Figure S1: Manipulation checks

Figure S2: Differences between the perceived control classes in self-report measures

Figure S3: Neural response to the ScanSTRESS-C across classes

Figure S4: ROI-analysis: Class difference in vmPFC activation under psychosocial stress

Table S1: Correlations of internal and external LoC with stress outcomes and mental health.

Table S2: Significant clusters for stress effect in the ScanSTRESS-C

Table S3: Clusters differing significantly between the classes in the ScanSTRESS-C

Table S4: Clusters significantly correlating with class probability in the ScanSTRESS-C

## Data Availability

The anonymized datasets generated and analyzed during the current study, as well as the computer code generated for the analyses are publicly available on the open science framework under this link: https://osf.io/g6au5/overview. Due to data protection, the neuroimaging data cannot be shared publicly but are available on request from the corresponding author (MW) and after filling out a research agreement.
