# Supplementary material for: Perceived control as a resilience factor: associations with neural, physiological and affective stress responses and mental health
Source: Transl Psychiatry. 2026 Jan 15;16:39. doi: 10.1038/s41398-025-03786-6 (PMC12824378; doi:10.1038/s41398-025-03786-6)
Supplement: Supplementary file 1 — Table of Contents of Supplementary Information [file 41398_2025_3786_MOESM1_ESM.pdf]

Table of Contents of Supplementary Information

Supplementary information accompanying the article "Perceived control as a resilience factor: Associations with neural, physiological and affective stress responses and mental health" by J. Meier, B. Kollmann, L. E. Meine, B. Meyer, K. Yuen, M. Stork, O. Tüscher & M. Wessa is available at TP's website.

Table S1: Correlations of internal and external LoC with stress outcomes and mental health.

Shows the correlations of all the outcomes reported in the paper with locus of control (LoC). No correlations are significant, indicating that perceived control classes better predict stress outcomes and mental health than LoC. PDF-file.

Table S2: Significant clusters for stress effect in the ScanSTRESS-C

Shows significant clusters activated and deactivated under the Stress vs. noStress conditions of the ScanSTRESS-C, aggregated across classes in the whole sample. PDF-file.

Table S3: Clusters differing significantly between the classes in the ScanSTRESS-C

Shows clusters that significantly differ between the classes in the contrast Stress vs. noStress and the inverse contrast. Three clusters emerged. The low-control class shows increased activation of the bilateral posterior insula and the primary motor cortex under psychosocial stress. PDF-file.

Table S4: Clusters significantly correlating with class probability in the ScanSTRESS-C

Results from the control analysis with class probability instead of class membership as predictor, to account for classification uncertainty. Clusters of activation significantly correlating with the probability to belong to the low-control class for the contrast stress > noStress. MNI = Montreal Neurological Institute, FWE = whole-brain family-wise error corrected on cluster-level.

Figure S1: Manipulation checks

Shows changes in self-reported affect and cortisol over the course of the experimental manipulations on testing days 1+2 for all participants (aggregated across perceived control classes). PDF-file.

Figure S2: Differences between the perceived control classes in self-report measures

Plots of all outcomes included into the self-report MANOVA by perceived control class. Errorbars denote bootstrapped 95% confidence intervals. n.s.: non-significant, * pHolm < .05, ** pHolm < .01, *** pHolm < .001. PDF-file.

Figure S3: Neural response to the ScanSTRESS-C across classes

Significant clusters for the effects of stress (a) and no stress (b) in the ScanSTRESS-C aggreagated across perceived control classes. Activation maps are thresholded at p=.05 FWE-corrected on peak-level and overlayed onto SPM152 template. PDF-file.

Figure S4: ROI-analysis: Class difference in vmPFC activation under psychosocial stress

Significant clusters (a) and parameter estimates at peak voxels of significant clusters (b) for the comparison of the low- and high-control class for the contrast stress>noStress. The region of interest was defined as a 16x20x20mm box at the MNI-coordinates 0 36 -8 extracted from a previous study that showed increased activation during controllable vs. uncontrollable stressors. Unexpectedly, we see increased activation in the low-control class. The parameter estimates indicate that as for the insula cluster, the low class didn’t discriminate between the noStress and stress conditions. Activation maps are thresholded at p=.05 FWE-corrected on cluster-level for the search volume (small volume correction) and overlayed onto the SPM152 template. ROI: region of interest, vmPFC: ventromedial prefrontal cortex, MNI: Montréal Neurological Institute, FWE: Family-wise error, *** p < .001. PDF-file.
