# Supplementary figures and images for: Perceived control as a resilience factor: associations with neural, physiological and affective stress responses and mental health
Source: Transl Psychiatry. 2026 Jan 15;16:39. doi: 10.1038/s41398-025-03786-6 (PMC12824378; doi:10.1038/s41398-025-03786-6)

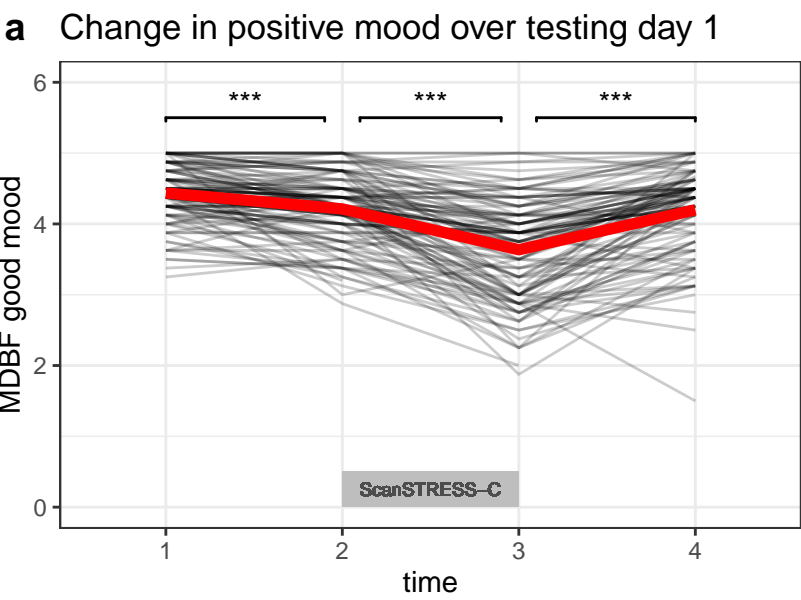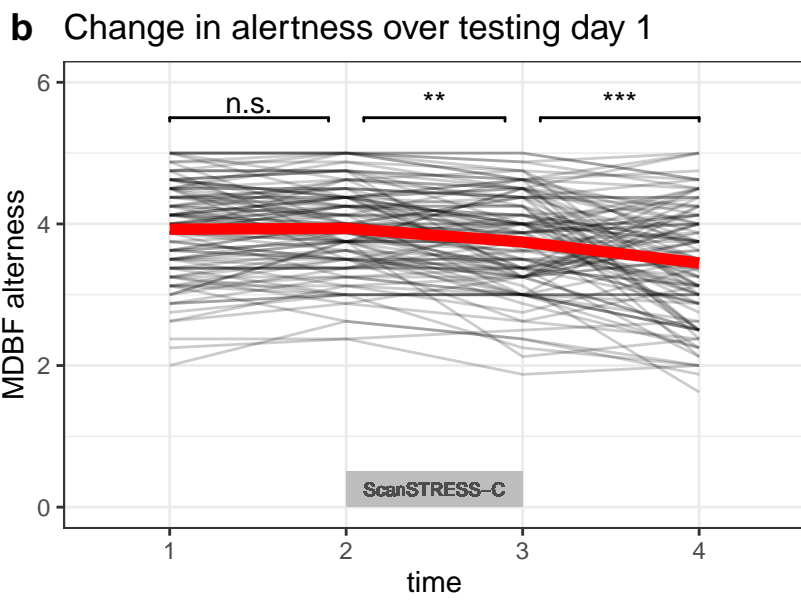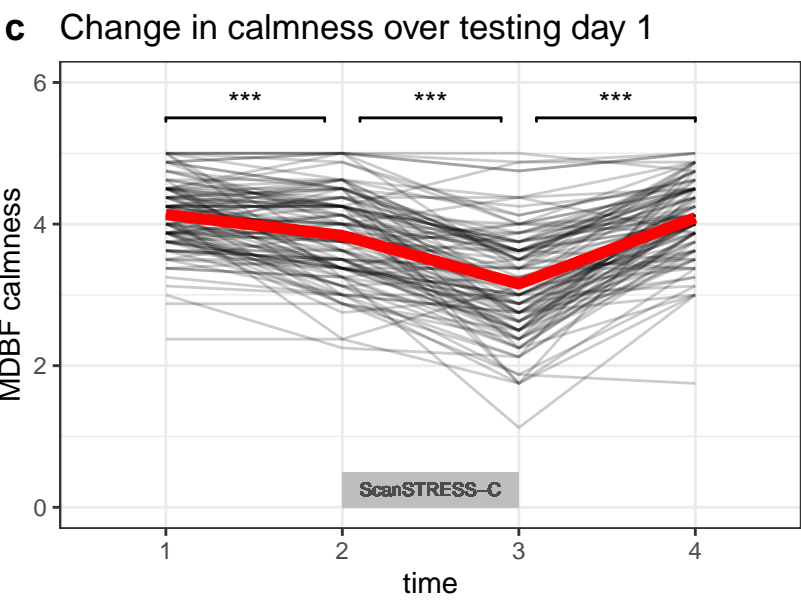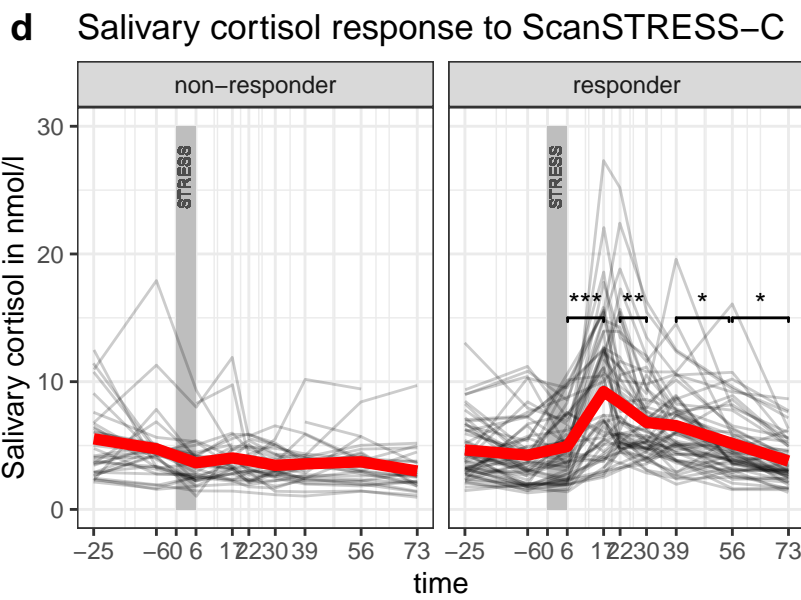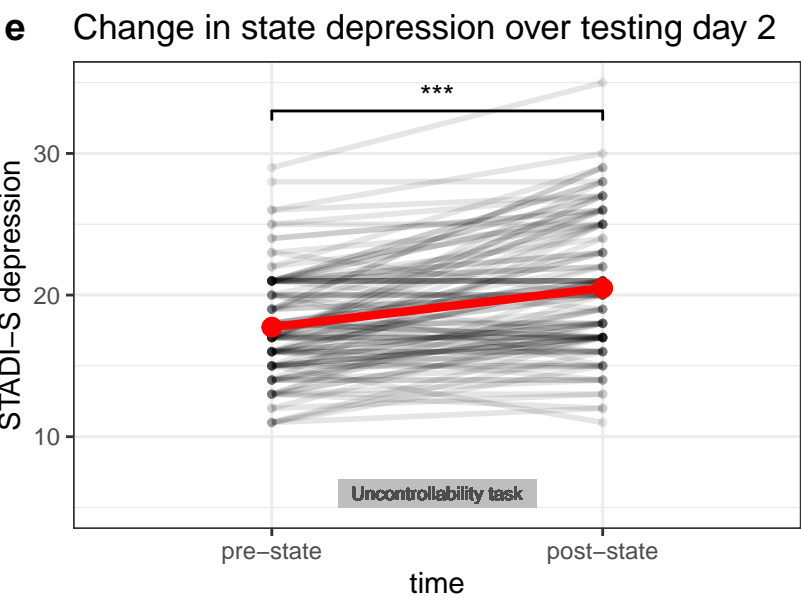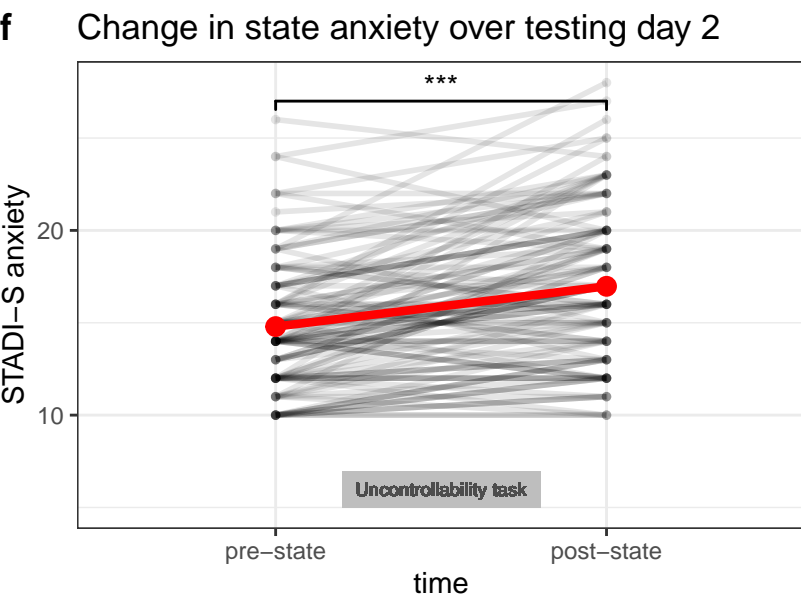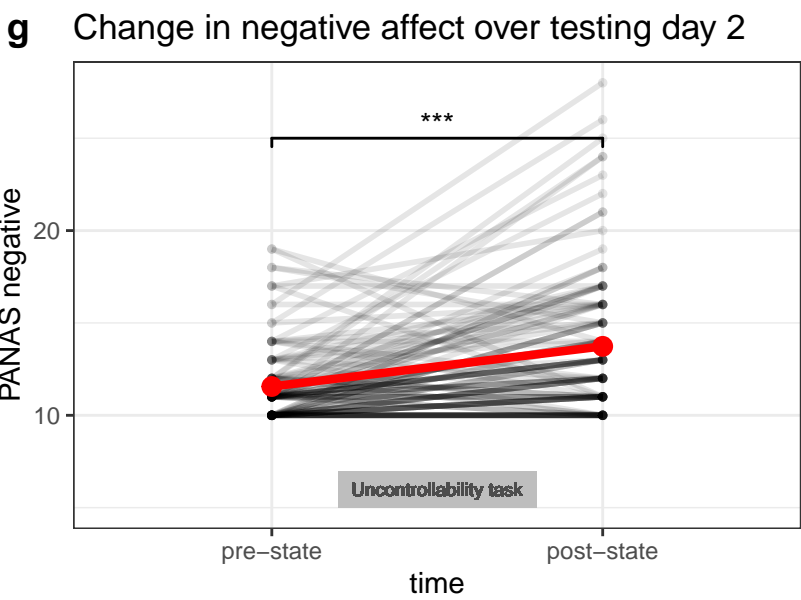

Supplement: Supplementary file 2 — Figure S1: Manipulation checks [file 41398_2025_3786_MOESM2_ESM.pdf]

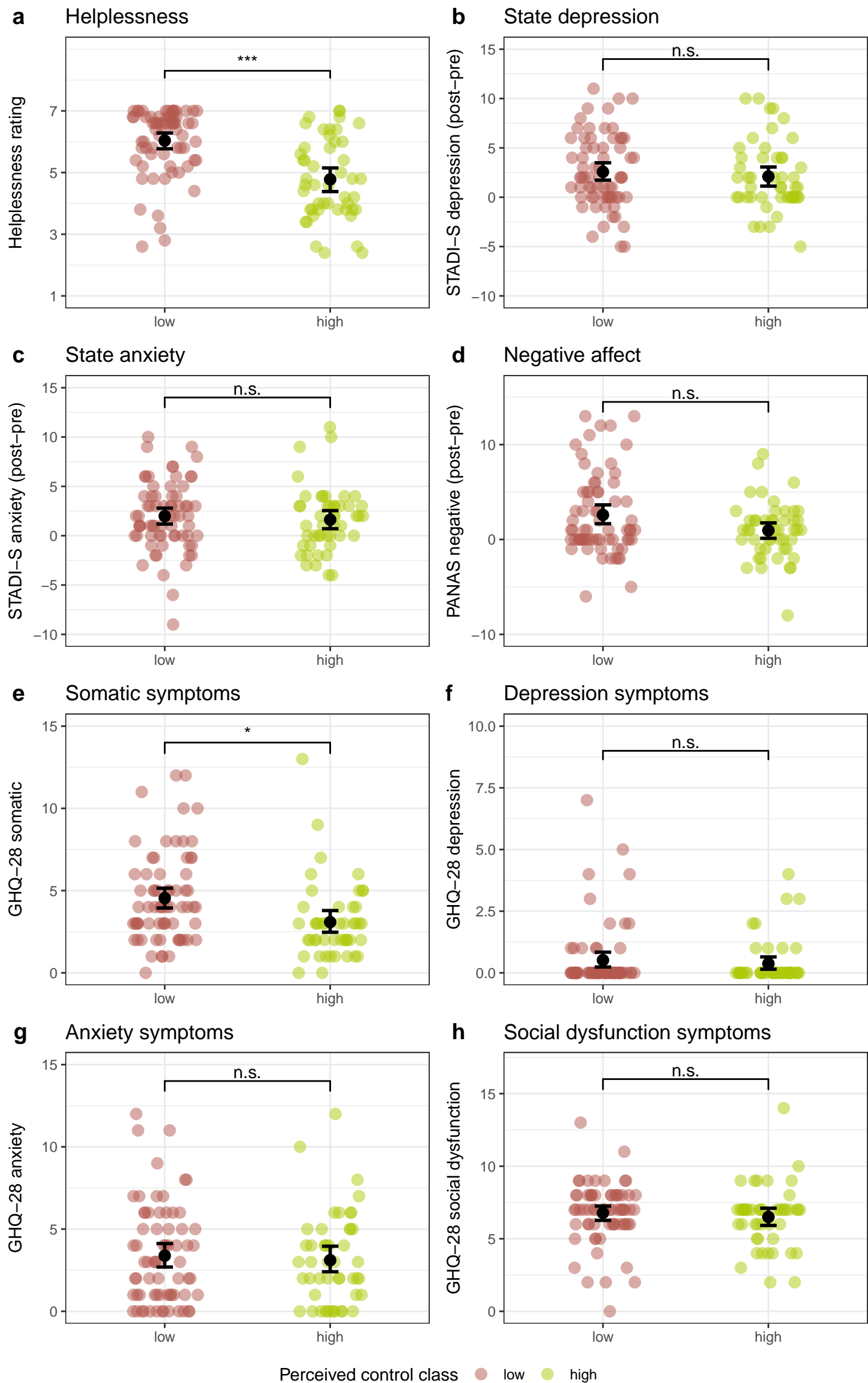

Supplement: Supplementary file 3 — Figure S2: Differences between the perceived control classes in self-report measures [file 41398_2025_3786_MOESM3_ESM.pdf]

**a. Stress > noStress**

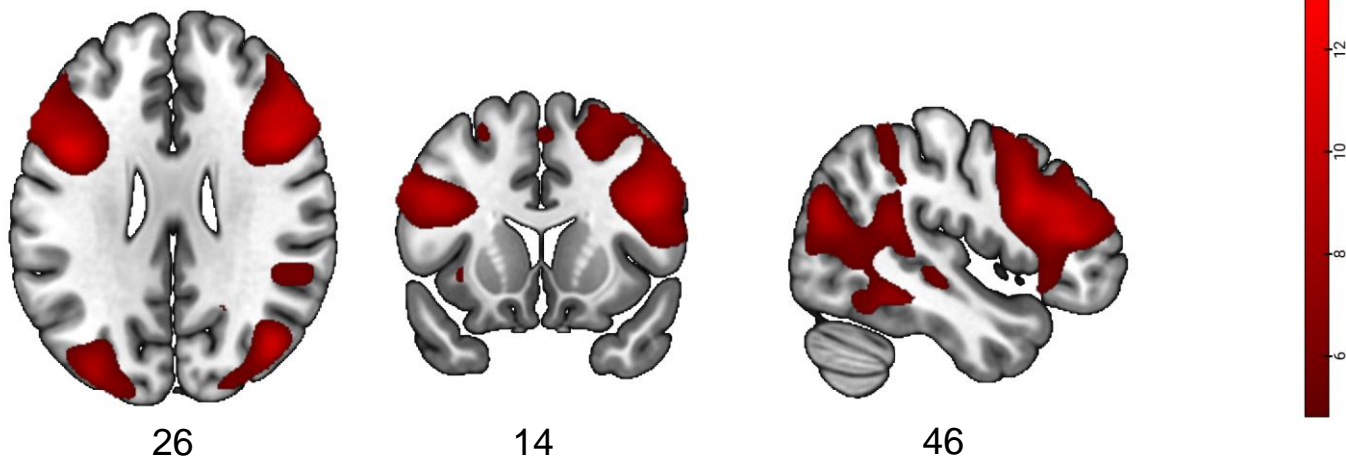

**b. noStress > Stress**

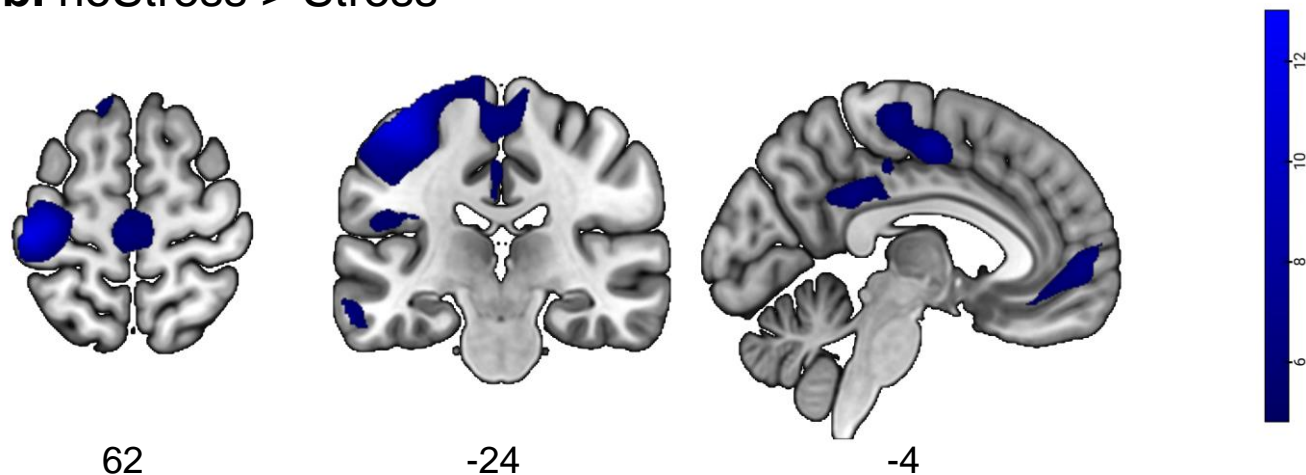

Supplement: Supplementary file 4 — Figure S3: Neural response to the ScanSTRESS-C across classes [file 41398_2025_3786_MOESM4_ESM.pdf]

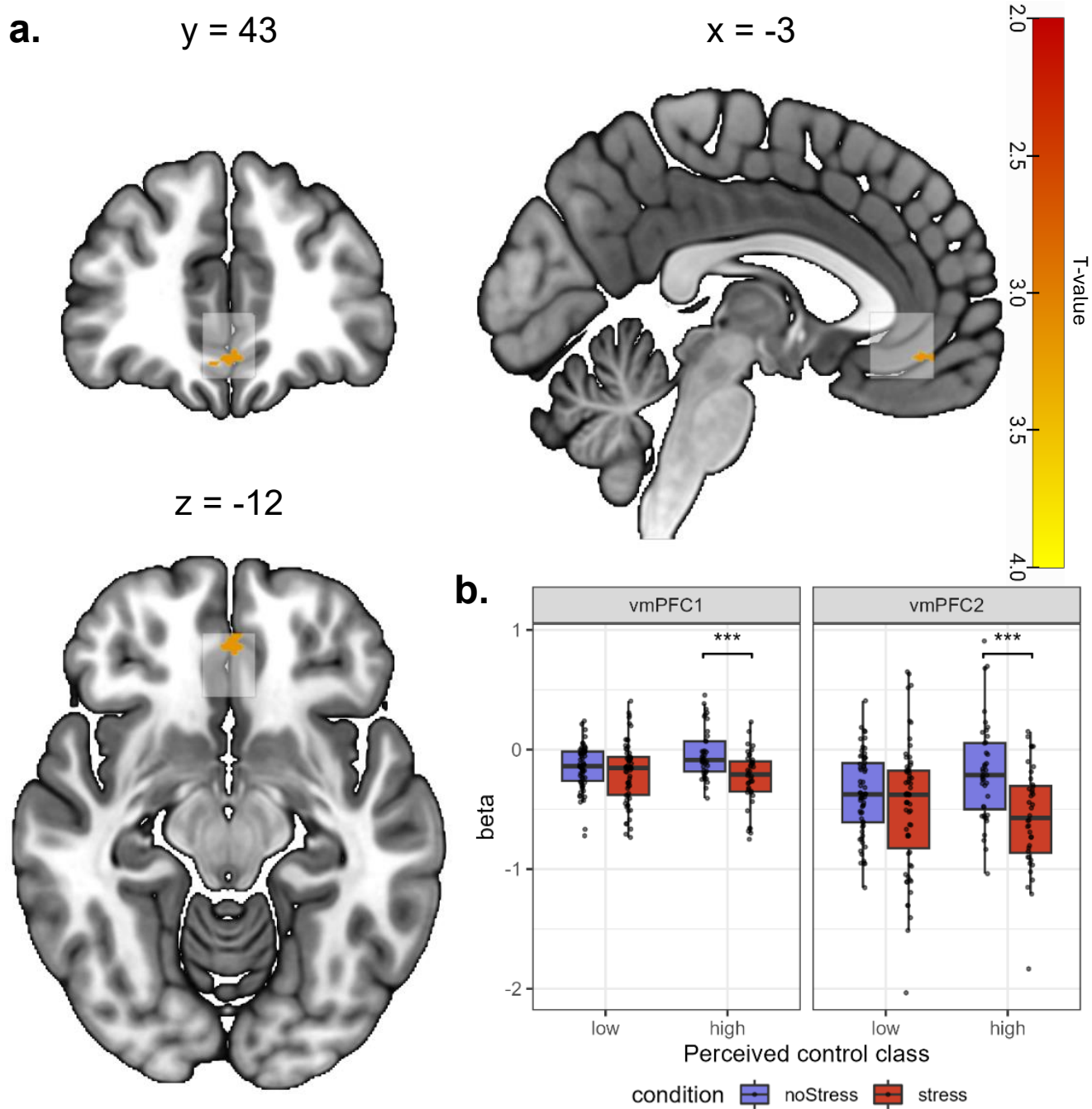

Supplement: Supplementary file 5 — Figure S4: ROI-analysis: Class difference in vmPFC activation under psychosocial stress [file 41398_2025_3786_MOESM5_ESM.pdf]
