# Supplementary material for: Perceived control as a resilience factor: associations with neural, physiological and affective stress responses and mental health
Source: Transl Psychiatry. 2026 Jan 15;16:39. doi: 10.1038/s41398-025-03786-6 (PMC12824378; doi:10.1038/s41398-025-03786-6)
Supplement: Supplementary file 6 — Table S1: Correlations of internal and external LoC with stress outcomes and mental health. [file 41398_2025_3786_MOESM6_ESM.pdf]

Table S1:

Correlations of internal and external LoC with stress outcomes and mental health

		Internal LoC		External LoC	
	N	*r*	*p*	*r*	*p*
Helplessness	114	0.13	>.999	0.05	>.999
STADI-S depression	114	0.03	>.999	0.00	>.999
STADI-S anxiety	114	-0.18	>.999	0.19	>.999
PANAS negative	114	0.02	>.999	0.09	>.999
GHQ28 depression	114	-0.23	.225	0.25	.168
GHQ28 anxiety	113	-0.20	.678	0.10	>.999
GHQ28 somatic	113	-0.10	>.999	0.27	.086
GHQ28 social dysfunction	114	-0.04	>.999	0.03	>.999
Cortisol AUC_i	84	0.04	>.999	-0.12	>.999
Cortisol AUC_g	84	-0.07	>.999	-0.10	>.999
Cortisol recovery	93	-0.02	>.999	-0.10	>.999

Note. Associations of internal and external Locus of Control (LoC) with self-report and endocrine stress outcomes and mental health. *p*-values are Holm-corrected. STADI-S: State-trait anxiety and depression inventory – state version. GHQ28: General Health Questionnaire, 28 item version. AUC_i: Area under the curve with respect to increase, AUC_g: Area under the curve with respect to ground.
