# Supplementary material for: Perceived control as a resilience factor: associations with neural, physiological and affective stress responses and mental health
Source: Transl Psychiatry. 2026 Jan 15;16:39. doi: 10.1038/s41398-025-03786-6 (PMC12824378; doi:10.1038/s41398-025-03786-6)
Supplement: Supplementary file 7 — Table S2: Significant clusters for stress effect in the ScanSTRESS-C [file 41398_2025_3786_MOESM7_ESM.pdf]

Table S2:

Significant clusters for the stress effect in the ScanSTRESS-C

Region		MNI coordinates			*T*	p^{FWE}	Voxels
		x	y	z			
**Stress > NoStress**							
Inferior frontal gyrus							
(pars opercularis)	L	-42	10	28	11.58	<.001	3081
	R	46	14	26	11.07	<.001	6180
Middle occipital gyurs	R	42	-72	26	10.76	<.001	15286
Thalamus	R	6	-26	-6	10.03	<.001	1205
Middle frontal gyrus	L	-22	8	54	7.77	<.001	318
	R	34	56	0	5.19	.001	58
Cerebellum 9	L	-12	-46	-48	7.51	<.001	91
Superior frontal gyrus							
medial part	R	4	30	48	7.00	<.001	423
orbital part	R	22	50	-12	6.54	<.001	114
Precuneus	R	18	-54	20	6.60	<.001	128
Superior temporal gyrus	R	50	-20	-6	6.42	<.001	145
Supramarginal gyrus	L	-46	-38	32	5.33	.010	14
**NoStress > Stress**							
Postcentral gyrus	L	-44	-24	62	12.04	<.001	2865
Angular gyrus	L	-52	-64	40	10.50	<.001	552
Putamen	L	-18	6	-6	10.24	<.001	988
	R	16	8	-8	8.47	<.001	294
Cerebellum 8	R	18	-58	-48	7.96	<.001	75
Cerebral crus 1	R	36	-78	-34	7.75	<.001	121
Superior frontal gyrus	L	-14	44	44	7.29	<.001	729
medial part	L	-6	56	4	6.78	<.001	339
Middle temporal gyrus	L	-60	-20	-20	6.51	<.001	160
Posterior cingulate	L	-2	-40	32	6.44	<.001	329
Cerebellum 4 5	R	18	-48	-22	6.07	.002	39
Precentral gyrus	R	36	-14	68	5.56	.007	20
Superior temporal gyrus	R	56	-2	0	5.22	.014	10
	L	-56	-10	6	5.19	.010	15

Note. Significant clusters with ≥ 10 voxels for the 1-sample *t*-tests stress>noStress and noStress>Stress across the whole sample, independent of perceived control class, anatomical labels derived with local maxima labelling using SPM toolbox AAL2, FWE = whole-brain family-wise error corrected on voxel-level
