# Supplementary material for: Perceived control as a resilience factor: associations with neural, physiological and affective stress responses and mental health
Source: Transl Psychiatry. 2026 Jan 15;16:39. doi: 10.1038/s41398-025-03786-6 (PMC12824378; doi:10.1038/s41398-025-03786-6)
Supplement: Supplementary file 8 — Table S3: Clusters differing significantly between the classes in the ScanSTRESS-C [file 41398_2025_3786_MOESM8_ESM.pdf]

Table S3

Clusters significantly differing between the classes in the ScanSTRESS-C

Region		MNI coordinates			*T*	p^{FWE}	voxels
		X	Y	Z			
**Stress > NoStress**							
Insula	R	40	-20	16	4.44	.011	409
Postcentral gyrus	R	40	-20	62	4.28	<.001	777
Insula	L	-36	-20	16	4.27	.007	455
**NoStress > Stress**							
			No suprathreshold clusters				

Note. Clusters of activation significantly differing between the perceived control classes for the contrast stress > baseline. MNI = Montreal Neurological Institute, FWE = whole-brain family-wise error corrected on cluster-level.
