# Supplementary material for: Perceived control as a resilience factor: associations with neural, physiological and affective stress responses and mental health
Source: Transl Psychiatry. 2026 Jan 15;16:39. doi: 10.1038/s41398-025-03786-6 (PMC12824378; doi:10.1038/s41398-025-03786-6)
Supplement: Supplementary file 9 — Table S4: Clusters significantly correlating with class probability in the ScanSTRESS-C [file 41398_2025_3786_MOESM9_ESM.pdf]

Table S4

Clusters significantly correlating with class probability in the ScanSTRESS-C

Region		MNI coordinates			*T*	p^{FWE}	voxels
		X	Y	Z			
**Stress > NoStress**							
Insula	R	40	-20	16	4.93	.001	741
Frontal superior gyrus	R	40	-20	62	4.55	<.001	1161
Insula	L	-40	-20	16	4.51	<.001	1089
**NoStress > Stress**							
			No suprathreshold clusters				

Note. Results from the control analysis with class probability instead of class membership as predictor, to account for classification uncertainty. Clusters of activation significantly correlating with the probability to belong to the low-control class for the contrast stress > noStress. MNI = Montreal Neurological Institute, FWE = whole-brain family-wise error corrected on cluster-level.
